# Frequency of recurrent injuries in the deciduous dentition and associated factors

**DOI:** 10.1590/1807-3107bor-2025.vol39.093

**Published:** 2025-09-08

**Authors:** Nathália Thaíse de Jesus OLIVEIRA, Patrícia SANTOS-SILVA, Izabella Barbosa FERNANDES, Cristiane Meira ASSUNÇÃO, Fernanda de Morais FERREIRA, Patrícia Maria ZARZAR, Raquel Gonçalves VIEIRA-ANDRADE

**Affiliations:** (a) Universidade de São Paulo – USP, School of Dentistry of Ribeirão Preto, Department of Pediatric Dentistry, Ribeirão Preto, SP, Brazil.; (b) Universidade Federal de Minas Gerais – UFMG, School of Dentistry, Department of Child and Adolescent’s Oral Health, Belo Horizonte, MG, Brazil.

**Keywords:** Tooth, Deciduous, Tooth Injuries, Child

## Abstract

Understanding recurrent injuries in the deciduous dentition and possible associated factors could help in the control and prevention of such episodes in children. The aim of the present study was to investigate the frequency of recurrent injuries in the deciduous dentition and associated factors. A retrospective cross-sectional study was conducted involving 517 children aged between six months and six years treated at the Clinic for Traumatic Dental Injuries in the Deciduous Dentition of the School of Dentistry of the *Universidade Federal de Minas Gerais*. Data were collected from dental records with information on sociodemographic and clinical characteristics. Data analysis involved descriptive statistics, chi-square test, and bivariate and multivariate Poisson regression analyses (p < 0.05; 95%CI). The prevalence of recurrent tooth injuries in the deciduous dentition was 17.2% (n = 89). Children aged between four and six years (PR = 1.917; 95%CI: 1.055–3.484; p = 0.033), those with inadequate lip protection (PR = 1.790; 95%CI: 1.085–2.953; p = 0.023), those with uncomplicated crown and crown-root fractures (PR = 1.856; 95%CI: 1.224–2.816; p = 0.004), and those with complicated crown and crown-root fractures (PR = 1.818; 95%CI: 1.021–3.239; p = 0.042) had a greater frequency of repeated tooth injuries compared to children without these characteristics. In the present study, older age, inadequate lip coverage, and the occurrence of complicated and uncomplicated crown and crown-root fractures were associated with repeated injuries in the deciduous teeth.

## Introduction

Traumatic dental injuries in deciduous teeth are one of the most common public health problems, with a prevalence of around 23% worldwide^1-3^ and 26% in Brazil.^4^ The cost of treating such injuries is high and has consequences for children’s oral health.^5,6^ The most common injuries in deciduous dentition are enamel fractures and dental luxation.^1,7^ Correct diagnosis and treatment are essential for a favorable long term prognosis.^8^

Preschool children with previous tooth injuries are at greater risk of further injury,^9^ which hampers the biological repair response of the affected tooth and related structures. Often, both the child and parents/guardians cannot recall a previous injury, which is detected in the radiographic exam and, with the new injury, makes the prognosis less favorable. Several sequelae to the deciduous teeth pulp and periodontium may be present (e.g., hyperemia, hemorrhage, necrosis, calcification, polyp, internal resorption of the crown and/or root and discoloration, external root resorption with or without infection, increase in the pericementary space, tooth mobility, displacement, ankylosis, alveolysis and prolonged retention). In addition, permanent teeth may also be affected due to the proximity of the deciduous tooth apex to the germ of the permanent successor.^4,10^

Few studies have evaluated the factors associated with recurrent injuries in deciduous dentition. As dental trauma causes pain and has unfavorable consequences for the deciduous and permanent dentition, especially in children with recurrent injuries, a better understanding of the subject is essential for guiding public health actions aimed at prevention and helping dentists in the counseling of parents and guardians.

Therefore, the aim of the present study was to investigate the frequency and associated factors of recurrent injuries in the primary dentition.

## Methods

A retrospective cross-sectional study was conducted at the Clinic of Traumatic Dental Injuries in the Deciduous Dentition of the School of Dentistry of the Universidade *Federal de Minas Gerais* (UFMG), a public university. This study received approval from the Human Research Ethics Committee of the university (protocol number: 3.386.630).

Data were collected from the dental records of 732 patients treated at the clinic, which is a reference center in the treatment of children with traumatic dental injures in the deciduous dentition in the city of Belo Horizonte, Brazil. Data on clinical and radiographic examinations were collected by undergraduate dental students who had undergone prior theoretical training on the criteria for identifying dental trauma as described by Andreasen et al.^11^ Andreasen’s classification has been accepted by the World Health Organization (WHO) and is recommended for use in data collection.^12^ The final diagnosis of all traumatic injuries was established after correlating clinical and radiographic findings. Additionally, the students were supervised by professors with extensive experience in the diagnosis and management of dental trauma in the primary dentition. A previously trained researcher collected the information from the dental records, which was transferred to a database. The records of children aged six months to six years treated between 2007 and 2019 (n = 517) were included. Records with missing data were excluded (29.3%, n = 215).

The patient records contained information obtained through interviews with parents/guardians on the socioeconomic status of the family, characteristics of the child, and information from the clinical and radiographic examination of the child for the identification of traumatic dental injuries and their clinical characteristics.

The outcome of this study (recurrent injuries in deciduous dentition) was considered when the child experienced at least one new dental trauma. Therefore, after returning to the clinic, the new trauma was confirmed through clinical and radiographic examinations and dichotomized as ‘yes’ or ‘no.

Non-clinical variables included sociodemographic conditions such as the mother’s schooling (years of schooling of more than 11, between 9 and 11, or up to 8) and monthly family income (up to 2 minimum wages or more than 2 minimum wages). Dental injury characteristics included immediate care (no or yes) and company at the moment of injury (parents/guardians or other/alone). Child characteristics included gender (girl or boy) and age (up to 2 years old, between 2 and 3 years old, between 3 and 4 years old, or between 4 and 6 years old). Age was divided into quartiles for better identifying potential variations in dental trauma across age groups. The clinical variables assessed included occlusal characteristics: severe overjet (no or yes) and lip protection (adequate or inadequate); type of traumatic dental injury: hard tissue injury (absent, uncomplicated crown fracture/crown-root fracture, or complicated crown fracture/crown-root fracture), soft tissue injury (absent or present), and injury to supporting tissue (absent or present); and injury characteristics: number of affected teeth (none/one or two or more) and tooth mobility (absent or present).

The data were entered into a databank and analyzed with the Statistical Package for the Social Sciences (SPSS 21.0). Descriptive analysis (absolute and relative frequencies) was performed for all variables. Data were also analyzed by the chi-square test (p ≤ 0.05) and bivariate and multivariate Poisson regression analyses. All variables with a p-value ≤ 0.25 in the bivariate analysis were included into the multivariate model and those with a p-value ≤ 0.05 (95%CI) after the adjustments were considered to be significantly associated with the outcome.

## Results

Of the children included in the study (n = 517), 59.2% were boys and 28.4% were aged between two and three years. The mean age was 37.69 months (± 14.7). The prevalence of recurrent tooth injuries was 17.2% (n = 89), with the upper central incisors being the most affected teeth in 53.9% of cases (n = 48). Most mothers had between 9 and 11 years of schooling (57.4%) and most families (77.6%) had a monthly income of more than 2 minimum wages (Table 1).

**Table 1:** Frequency distribution of the children attended (n = 517) according to study variables.

Variables	n (%)
Dependent variable	
Recurrent injury	
No	428 (82.8)
Yes	89 (17.2)
Independent variables	
Non clinical variables	
Characteristics of traumatic dental injuries	
Seeking immediate care	
No	274 (53.0)
Yes	243 (47.0)
Persons at the moment of traumatic dental injury	
Parents/Guardians	296 (57.3)
Other/ Alone	221 (42.7)
Child characteristics	
Gender	
Girls	211 (40.8)
Boys	306 (59.2)
Age	
Up to 2 years old	111 (21.5)
Between 2 and 3 years old	147 (28.4)
Between 3 and 4 years old	131 (25.3)
Between 4 and 6 years old	128 (24.8)
Sociodemographic conditions	
Mother’s schooling	
More than 11 years	136 (26.3)
Between 9 and 11 years	297 (57.4)
Up to 8 years	84 (16.2)
Monthly family income	
Up to 2 minimum wages	401 (22.4)
More than 2 minimum wages	116 (77.6)
Clinical variables	
Occlusion characteristics	
Severe overjet (greater than 3mm)	
No	456 (88.2)
Yes	61 (11.8)
Lip protection	
Adequate	472 (91.3)
Inadequate	45 (8.7)
Types of traumatic dental injuries	
Hard tissue injuries	
Absent	239 (46.2)
Uncomplicated crown fracture/crown-root fracture	213 (41.2)
Complicated crown fracture/ crown-root fracture	65 (12.6)
Soft tissue injuries	
Absent	305 (59.0)
Present	212 (41.0)
Supporting tissue injuries	
Concussion/Subluxation	
Absent	426 (82.4)
Present	91 (17.6)
Luxation (lateral, intrusion and extrusion)	
Absent	332 (64.2)
Present	185 (35.8)
Avulsion	
Absent	440 (85.1)
Present	77 (14.9)
Characteristics of traumatic dental injuries	
Number of teeth affected	
None/one	189 (36.6)
Two or more	328 (63.4)
Tooth mobility	
Absent	286 (55.3)
Present	231 (44.7)

Among the types of injury, the prevalence of soft tissue injuries (n = 212), hard tissue injuries (n = 278), and supporting tissue injuries (n= 353) was 41%, 53.8%, and 68.3% respectively. A total of 63.4% of the sample (n = 328) had injuries in two or more teeth (Table 1).

Table 2 displays the associations between recurrent tooth injuries and the independent variables of interest. Statistically significant associations were found with age between four and six years (p = 0.036), inadequate lip protection (p = 0.030), hard tissue injury (p = 0.032), and having two or more teeth involved in the injury (p = 0.021).

**Table 2:** Association between recurrent tooth injuries in the deciduous dentition and the independent variables (n = 517).

Variables	Repeated injuries			
	No (%)	Yes (%)	Total (%)	p-value
Non clinical variables				
Characteristics of traumatic dental injuries				
Immediate care				
No	228 (83.2)	46 (16.8)	274 (100.0)	
Yes	200 (82.3)	43 (17.7)	243 (100.0)	0.785*
Person at the moment of injury				
Parents/Guardians	250 (84.5)	46 (15.5)	296 (100.0)	
Other/ Alone	178 (80.5)	43 (19.5)	221 (100.0)	0.243*
Child characteristics				
Gender				
Girls	178 (84.4)	33 (15.6)	211 (100.0)	
Boys	250 (81.7)	56 (18.3)	306 (100.0)	0.431*
Age				
Up to 2 years old	98 (88.3)	13 (11.7)	111 (100.0)	
Between 2 and 3 years old	123 (83.7)	24 (16.3)	147 (100.0)	
Between 3 and 4 years old	107 (81.7)	24 (18.3)	131 (100.0)	
Between 4 and 6 years old	100 (78.1)	28 (21.9)	128 (100.0)	0.036***
Sociodemographic conditions				
Mother’s schooling				
More than 11 years	114 (83.8)	22 (16.2)	136 (100.0)	
Between 9 and 11 years	247 (83.2)	50 (16.8)	297 (100.0)	
Up to 8 years	67 (79.8)	17 (20.2)	84 (100.0)	0.715*
Monthly family income				
Up to 2 minimum wages	330 (82.3)	71 (17.7)	401 (100.0)	
More than 2 minimum wages	98 (84.5)	18 (15.5)	116 (100.0)	0.582*
Clinical variables				
Occlusion characteristics				
Severe overjet (greater than 3mm)				
No	378 (82.9)	78 (17.1)	456 (100.0)	
Yes	50 (82.0)	11 (18.0)	61 (100.0)	0.857*
Lip protection				
Adequate	396 (83.9)	76 (16.1)	472 (100.0)	
Inadequate	32 (71.1)	13 (28.9)	45 (100.0)	0.030*
Types of traumatic dental injuries				
Hard tissue injuries				
Absent	209 (87.4)	30 (12.6)	239 (100.0)	
Uncomplicated crown fracture/crown-root fracture	167 (78.4)	46 (21.6)	213 (100.0)	
Complicated crown fracture/ crown-root fracture	52 (80.0)	13 (20.0)	65 (100.0)	0.032*
Soft tissue injuries				
Absent	251 (82.3)	54 (17.7)	305 (100.0)	
Present	177 (83.5)	35 (16.5)	212 (100.0)	0.723*
Supporting tissue injuries				
Concussion/Subluxation				
Absent	357 (83.8)	69 (16.2)	426 (100.0)	
Present	71 (78.0)	20 (22.0)	91 (100.0)	0.185*
Luxation (lateral, intrusion and extrusion)				
Absent	270 (81.3)	62 (18.7)	332 (100.0)	
Present	158 (85.4)	27 (14.6)	185 (100.0)	0.239*
Avulsion				
Absent	363 (82.5)	77 (17.5)	440 (100.0)	
Present	65 (84.4)	12 (15.6)	77 (100.0)	0.681*
Characteristics of traumatic dental injuries				
Number of teeth that had suffered injury				
None/one	166 (87.8)	23 (12.2)	189 (100.0)	
Two or more	262 (79.9)	66 (20.1)	328 (100.0)	0.021*
Tooth mobility				
Absent	240 (83.9)	46 (16.1)	286 (100.0)	
Present	188 (81.4)	43 (18.6)	231 (100.0)	0.449*

*Chi-square test (p £ 0.05); ** Linear association (p £ 0.05).

In the final adjusted multivariate model, children aged four to six years (PR = 1.917; 95%CI: 1.055–3.484; p = 0.033), those with inadequate lip protection (PR = 1.790; 95% CI: 1.085–2.953; p = 0.023), those with uncomplicated crown and/or crown-root fractures (PR = 1.856; 95%CI: 1.224–2.816; p = 0.004), and those with complicated crown and/or crown-root fractures (PR = 1.818; 95%CI: 1.021–3.239; p = 0.042) had a greater frequency of repeated tooth injuries compared to children without these characteristics (Table 3).

**Table 3:** Poisson regression model for the association between recurrent injuries in the deciduous dentition and the independent variables (n =517).

Variables	Non-adjusted	p-value	Adjusted	p-value*
	(95%CI)		(95%CI)	
Non clinical variables				
Characteristics of traumatic dental injuries				
Immediate care				
No	1			
Yes	0.949 (0.650-1.385)	0.785	-	-
Person at the moment of injury				
Parents/Guardians	1			
Other/ Alone	1.252 (0.858-1.825)	0.243	-	-
Child characteristics				
Gender				
Boys	1			
Girls	0.855 (0.577-1.266)	0.433	-	-
Age				
Up to 2 years old	1		1	
Between 2 and 3 years old	1.394 (0.744-2.613)	0.300	1.335 (0.723-2.467)	**0.356**
Between 3 and 4 years old	1.564 (0.837-2.925)	0.161	1.633 (0.885-3.014)	**0.116**
Between 4 and 6 years old	1.868 (1.018-3.426)	**0.044**	1.917 (1.055-3.484)	**0.033**
Sociodemographic conditions				
Mother’s schooling				
More than 11 years	1			
Between 9 and 11 years	1.041 (0.658-1.646)	0.865	-	-
Up to 8 years	1.251 (0.706-2.216)	0.442	-	-
Monthly family income				
Up to 2 minimum wages	1			
More than 2 minimum wages	1.141 (0.710-1.833)	0.585	-	-
Clinical variables				
Occlusion characteristics				
Severe overjet (greater than 3mm)				
No	1			
Yes	1.054 (0.595-1.868)	0.856	-	-
Lip protection				
Adequate	1		1	
Inadequate	1.794 (1.085-2.966)	**0.023**	1.790 (1.085-2.953)	0.023
Types of traumatic dental injuries				
Hard tissue injuries				
Absent	1			
Uncomplicated crown fracture/crown-root fracture	1.721 (1.129-2.622)	**0.012**	1.856 (1.224-2.816)	0.004
Complicated crown fracture/ crown-root fracture	1.593 (0.883-2.875)	0.122	1.818 (1.021-3.239)	0.042
Soft tissue injuries				
Absent	1			
Present	0.932 (0.633-1.374)	0.724	-	-
Supporting tissue injuries				
Concussion/Subluxation				
Absent	1			
Present	1.357 (0.871-2.114)	0,177	-	-
Luxation (lateral, intrusion and extrusion)				
Absent	1			
Present	0.782 (0.516-1.183)	0.244	-	-
Avulsion				
Absent	1			
Present	0.891 (0.510-1.556)	0.684	-	-
Characteristics of traumatic dental injuries				
Number of teeth that had suffered injury				
None/one	1		1	
Two or more	1.653 (1.065-2.566)	**0.025**	1.524 (0.976-2.382)	0.064
Tooth mobility				
Absent	1			
Present	1.157 (0.793-1.689)	0.448	-	**-**

*Values adjusted for child’s age, lip protection, hard tissue injuries, and number of teeth affected.

## Discussion

The present study investigated the frequency of recurrent tooth injuries in the deciduous dentition and associations with characteristics of the child, sociodemographic aspects, and characteristics related to the tooth injury. As traumatic dental injuries can impact both the deciduous and permanent dentitions and teeth with recurrent injuries have a poorer prognosis,^4^ it is important to investigate factors associated with repeated injuries to prevent such events. The results of the present study showed that older age of the child, the presence of inadequate lip coverage and the occurrence of uncomplicated and complicated crown and crown-root fractures, which are hard tissue injuries, were associated with recurrent tooth injuries in the analyzed children.

The prevalence of recurrent tooth injuries in the deciduous dentition was 17.2%, which is close to rates reported in previous studies conducted in other reference centers, such as 16.7%^13^ and 16.9%.^14^ According to the literature, deciduous teeth that suffer several injuries can have greater complications, such as crown discoloration and fistulas,^15,16^ which can lead to tooth loss.

Age four to six years was associated with repeated tooth injuries compared to children up to two years of age. Tooth injuries are common in the preschool years, and the main cause is falls. According to Granville-Garcia,^17^ older children in this age group are more active and independent and are therefore more susceptible to traumatic dental injuries. It is also important to bear in mind that tooth injuries are cumulative, and children who suffer tooth injuries in early childhood are at greater risk of suffering further injuries in the future.^18^

Inadequate lip protection is reported to be one of the factors most strongly associated with traumatic dental injuries,^4,19-21^ which can be explained by the fact that lip sealing could partially absorb the impact of trauma, preventing dental fractures.^22^ A systematic review with meta-analysis^23^ involving 32 studies found an association between inadequate lip coverage and tooth injuries in the deciduous dentition and the combined effect had an odds ratio (OR) of 1.86 (95%CI: 1.24–2.79; n = 5680). This shows that inadequate lip protection makes the anterior teeth more vulnerable to traumatic dental injury, including recurrent injuries.

Children with uncomplicated and complicated crown and crown-root fractures had a 1.8-fold higher frequency of recurrent tooth injuries compared to children without such fractures. This suggests that the mere presence of a hard tissue injury, regardless of its severity, is a risk factor for the occurrence of further injuries in deciduous dentition, as demonstrated in a longitudinal study conducted with preschool children.^24^ It is important to note that in this study, the group of children without hard tissue fractures consisted of children who either had soft tissue injuries, less severe injuries, or no evident injuries when they were examined.

Family income was not associated with recurrent tooth injuries, in agreement with previous studies that found no association between dental trauma and socioeconomic conditions,^15,25-27^ including a prospective longitudinal study conducted in Brazil^24^and a systematic review with meta-analysis.^28^ Only one study reported a positive association between socioeconomic factors and injuries to deciduous teeth^29^. Comparisons between studies should be made with caution due to methodological differences, such as different indices, sample origins, age groups, and statistical analyses.^24,30^ In this study, monthly family income was assessed using a cutoff point of two monthly minimum wages, while other studies have used different cutoff points, such as three Brazilian minimum wages,^31^ values representative of the Brazilian minimum wage in other countries^32^ or specific indicators, such as the Social Vulnerability Index, which was developed exclusively for the city of Belo Horizonte, Minas Gerais, Brazil.^33^

Mother’s education also did not have an influence on recurrent tooth injuries in the present investigation. This finding is in agreement with data described in previous studies,^24-27,34^ but differs from cross-sectional and longitudinal investigations conducted with Brazilian children, in which traumatic dental injuries were associated with a higher level of mother’s schooling in some studies^35,36^ and a lower level in others.^33^ Such differences may be due to how this variable is categorized. In this study, mother’s schooling was categorized by tertiles, while in other studies this variable was dichotomized, using 8 years of schooling as the cut-off point.^31,35,36^

This study has some methodological limitations, such as the assessment of clinical conditions. Although the dental students received prior training, they were not trained and calibrated for clinical data collection, as is done in some epidemiological studies. However, this limitation was mitigated by the direct supervision of expert professors in the clinical evaluation and radiographic analysis of traumatized deciduous teeth the detection of dental trauma in deciduous dentition using the Andreasen Index.^11^ Another limitation refers to the number of missing data in the dental records, as this was a retrospective study.

The results of the present study are important for guiding public health actions aimed at the prevention of tooth injuries, including educational actions with teachers, employees, and parents/guardians. These findings can also help dental professionals in to advise parents and guardians regarding the importance of following up of all types of traumatic dental injuries in primary teeth until the eruption of the permanent successor. Such actions could help decrease the occurrence of recurrent tooth injuries in preschool children and their complications in the deciduous and permanent dentitions. Moreover, understanding the factors associated with such injuries allows dental professionals to provide better guidance on preventive measures, reducing the likelihood of further trauma and minimizing the severity of injuries to the same tooth. It is important that further studies, especially prospective studies, be conducted to deepen the understanding of these factors and optimize prevention strategies.

## Conclusion

Based on the present findings, older age (four to six years), inadequate lip coverage, and complicated and uncomplicated crown and crown-root fractures were associated with recurrent injuries in the deciduous teeth.

## Data Availability

The datasets generated during and/or analyzed during the current study are available from the corresponding author on reasonable request.
